# Nurses’ information exchange during older patient transfer: prevalence and associations with patient and transfer characteristics

**DOI:** 10.5334/ijic.879

**Published:** 2013-03-01

**Authors:** Rose Mari Olsen, Ove Hellzén, Ingela Enmarker

**Affiliations:** Faculty of Health and Science, Nord-Trøndelag University College, Finn Christiansens vei 1, No-7800 Namsos, Norway; Department of Health Sciences, Mid-Sweden University, S-851 70 Sundsvall, Sweden; Faculty of Health and Science, Nord-Trøndelag University College, Finn Christiansens vei 1, No-7800 Namsos, Norway; Centre for Care Research Mid-Norway, Servicebox 2501, NO-7729 Steinkjer, Norway

**Keywords:** older people, transfer, nursing documentation, hospitalization, home care

## Abstract

**Introduction:**

To ensure continuity of care, it is important to effectively communicate the health status of older patients who are transferred between health care organizations. The objectives of this study were to: (1) evaluate the prevalence of nursing transfer documents, and (2) identify patient and transfer characteristics associated with the presence of nursing transfer documents for older patients transferred from home care to hospital and back to home care again after hospitalization.

**Methods:**

Nursing documents were reviewed from a total of 102 records of older inpatients admitted from home care to medical wards at a local hospital in central Norway and later discharged home. Frequencies were used to describe patient and transfer characteristics, and the prevalence of transfer documents. Pearson’s χ^2^ test and logistic regression were used to identify possible associations between patient and transfer characteristics and the presence of nursing transfer documents.

**Results:**

While nursing admission notes were present in 1% of the patient transfers from home care to the hospital, 69% of patient discharges from the hospital to home care were accompanied by nursing discharge notes. Patient and transfer characteristics associated with the presence of a nursing discharge note were age, gender, medical department facility, and length of hospital stay.

**Conclusions:**

The low prevalence of nursing transfer documents constitutes a challenge to the continuity of care for hospitalized home care patients. Patient and transfer characteristics may impact the nurses’ propensity to exchange patient information. These findings emphasize the need for nurses and managers to improve the exchange of written information. While nurses must strive to transfer accurate patient information at the right place and at the right time, the managers must facilitate this by providing appropriate guidelines and standards, as well as adequate personnel and resources.

## Introduction

Published studies and government reports have pointed out that continuity of care is important and beneficial for older people [1–6]. Older people often have multiple co-morbidities and complex needs [7], they often experience a greater number of hospitalizations and outpatient visits [8], and for some of them, hospitalization is followed by an irreversible decline in functional status [9]. In situations of care transfer, knowledge of the older patient’s health status, as well as usual condition, and the recent stability of this condition are significant. Although processes and outcomes of care transfer might be influenced by the descriptions conveyed by the referring provider [10–12], many studies have reported inaccuracy and incompleteness in essential information during the transfer of older people [10, 13–16]. In Norway, promoting the coordination of health care, i.e., integrating activities between health care organizations to ensure appropriate service delivery, has been one of the top government priorities of the last decade [17]. The Norwegian health care system is divided into two organizational structures: primary care and secondary care. While primary care (including GP, nursing homes, and home care) is managed by local municipalities, the central government owns and runs the hospitals. The health authorities [18, 19] have stated that the lack of interaction between primary and secondary care represents perhaps the greatest challenge facing the health service. This paper reports on a study that is part of a larger research project concerning nurses’ information exchanges during the transfer of older people between home care (HC) and hospitals in Norway.

The fragmentation of health care, i.e., having multiple decision makers make a set of health care decisions that would be made better through unified decision-making [20: 1], has over the past years been a subject of worldwide concern [21–24]. There has been increasing focus on the importance of continuity of care in improving preventive care, fewer hospitalizations, and patient outcomes and satisfaction [3, 5, 25–28], and this has been accompanied by a discussion aimed at clarifying the concept of continuity of care [29, 30]. A widely used framework, introduced by Haggerty et al. [31] and later adopted by Freeman et al. [32], describes three essential types of continuity of care: relational, management, and informational. While the relational type concerns the relationship between a patient and one or more providers, the informational and management types deal with, respectively, the timely availability of relevant information to make current care appropriate for each individual, and the communication of both facts and judgments across providers, and between providers and patients [32]. It is argued that an effective healthcare organization has to embody all three types of continuity, alongside good access and systematic care [33]. The present study, however, is mainly concerned with the informational and management types of continuity.

According to the Norwegian Health Personnel Act [34], nurses are obligated to document care provided in the patient record and to exchange relevant and necessary information to ensure the continuity of care. In a separate regulation [35], the act requires that in cases of transfer from one health institution to another, a summary of the patient record shall be sent to health professionals who need the information in order to provide the patient with appropriate follow-up care. The nursing summary should include descriptions of the nursing care delivered, the patient’s status, and assessments and recommendations for continuing care [36, 37]. Today, most summaries exchanged between primary and secondary care providers are still paper-based [38].

Although nurses at hospitals and primary care facilities have contrasting perceptions of the extent of information exchange that occurs during patient transfer [39–41], studies concerning the transfer of written information between facilities have mostly focused on the quality of the content rather than its prevalence or availability [42, 43]. However, two studies reporting prevalence of nursing discharge notes have been found. In a Norwegian study of records of patients discharged from hospital to HC [44], 58% (n=21) of the records contained a nursing discharge note. In a study aimed to evaluate the effects of nursing documentation intervention on the quantity and quality of nursing documentation at a hospital in Sweden [45], only 5% (n=3) of the patient records contained a nursing discharge note before the intervention. The amount increased to 39% (n=23) after the 2-year intervention, and 53% (n=32) at the 3-year follow-up. There have been several studies that have addressed the prevalence of transfer documents between physicians in hospital and primary care [14, 46–50]. Most of these studies have been concerned with communication during discharge from the hospital [47–49]. A review [47] reported that approximately 25% of the summaries never reached the primary care physician. With regard to communication during hospital admission, studies have reported that 6–58% of the patients transferred lacked physician transfer documents [46, 50]. A previous study found that nursing homes were more likely to receive complete transfer information from hospitals that offered geriatric specialty care and had fewer hospital beds [51]. However, information gaps between hospital geriatric wards and primary care have also been reported [14].

To our knowledge, previous studies that have investigated the prevalence of transfer documents have primarily addressed communication between physicians. Furthermore, few studies have considered the entire communication loop during an episode of hospitalization, i.e., communication both at admission and discharge; instead, they have only focused on one-way transmission. It therefore appears to be important to study the whole process when older HC patients experience an episode of hospitalization. A better understanding of nurses’ exchanges of written patient information during the transfer of older people from HC-to-hospital-to-HC could support nurses in improving the continuity of care and promoting safe patient transfer. An optimal patient transfer is an important element in the continuum of care and for the integration of services between primary and secondary care.

### The aim of the study

The objectives of this study were to: (1) evaluate the prevalence of nursing transfer documents, and (2) identify patient and transfer characteristics associated with the presence of nursing transfer documents for older patients transferred from HC to hospital and back to HC again after hospitalization.

## Method

### Setting and participants

The data were collected at the medical department at a local hospital in central Norway during 2010–2011. Nursing documentation from inpatient records was collected using consecutive convenience sampling. The inclusion criteria were that the patients were 70+ years of age, consent competent, and admitted from HC and were discharged to their homes after the hospital treatment. In Norway, users of HC can receive home nursing care (e.g., assistance with personal hygiene, meals, wound care, and medication) and/or practical help (e.g., in-home cleaning and laundry services). In the current study, HC is defined as home nursing care, thus patients only receiving practical help from the HC were excluded. Since sheltered housing has no permanent staff and the resident may receive HC services as needed on an individual basis as other people living at home, both patients living in traditional homes and in sheltered housing were included. In order to ensure that participants were anonymous to the researchers, nursing managers at the respective units evaluated all patients for the inclusion criteria. A sample size of 100 participants was determined to be adequate [52]. Of 111 patients who met the inclusion criteria, 9 refused to participate. Thus the final sample size was 102, 70 from the general medicine ward and 32 from a geriatric unit. The nursing managers numbered the patient transfers consecutively.

### Data collection

The nursing managers were responsible for retrieving transfer documents and background data for patients who were included. They were instructed to delete all information that could identify the patients.

#### Transfer documents and background data

When patients were transferred between the hospital and HC, paper transfer documents were transported between facilities. The transfer documents were retrieved by the nursing managers on the day of discharge and were supposed to contain both an admission note (from HC to hospital) and a discharge note (from hospital to HC). Background data were used to identify patient characteristics (age, gender, living situation, housing situation, and distance from hospital) and transfer characteristics (type of hospitalization, readmission, medical department facility, and length of hospital stay). The nursing managers were instructed to obtain data about the patients’ gender, age, and readmission status (within 30 days after discharge). Nursing discharge planning notes were present for all the patients and became part of the background data. In addition, physicians’ discharge notes were usually attached to the nursing discharge notes because they contained medication information. These notes became background data as well.

### Data analysis

SPSS version 17.0 for Windows (SPSS, Inc., Chicago, Illinois, USA) was employed for statistical analysis. Frequencies were used to describe patient and transfer characteristics, and the prevalence of transfer documents. The bivariate relationships between the presence of transfer documents (dependent variable) and the patient and transfer characteristics (independent variables) were conducted using the Pearson’s χ^2^-test, and the odds ratio (OR) was calculated for each independent variable. We quantify the associations of patient and transfer characteristics with the presence of a nursing transfer document using logistic regression analysis (enter method). Causes for hospitalization were used to describe the sample; however, they were not used in the regression analysis due to small subsample size. Several models were considered in order to optimize significance and model quality before a final model was chosen. Confidence intervals (CI) of 95% were calculated for the OR. Model fit was measured with the Hosmer and Lemeshow goodness-of-fit test, and a test of multicollinearity (VIF) among covariates was performed to assess possible linear dependencies among covariates included in the model.

### Ethical considerations

The study was conducted in accordance with the Declaration of Helsinki [53], and the research was approved by The Committee for Medical and Health Research Ethics of Norway (nr 2009/815). Permission was obtained from the hospital research unit to perform data collection in the hospital wards. Informed consent was obtained from all patients. They were guaranteed confidentiality and assured that their anonymity would be preserved when the findings were presented. No identifying information about the patients or their next of kin was available to the researchers.

## Results

### Characteristics of hospitalized HC patients

A total of 102 hospitalizations of HC patients were included. Characteristics of the patients are shown in Table 1. The mean age was 82.9 years (range 70–95) and 57.8% were females. Most of the patients lived alone (69.6%) and in their own homes (72.5%). The most common causes of hospitalization were pneumonia (31.4%), chronic obstructive pulmonary disease (11.8%), anemia (8.8%), syncope (5.9%), repeated falls (5.9%), myocardial infarction (4.9%), congestive heart failure (2.9%), urinary tract infection (2.9%), and dyspnea (2.9%). Eighty-four percent of transfers were considered urgent. The median length of hospital stay was 6 days (range 1–29). Almost 25% of the transfers were hospital readmissions, within 30 days of return to the HC.

### Patient and transfer characteristics and presence of transfer documents

A nursing admission note was identified for 1 of the 102 patients (1%), a sample that is obviously too small to statistically calculate results, and therefore was not further investigated. As shown in Table 2, nursing discharge notes were identified for 70 patients (68.6%). The χ^2^-test detected a significant association between the presence of a discharge note and both medical department facility (p<0.001) and age (p<0.01) (Table 2). Calculations of the odds ratios for the presence of a discharge note due to medical department facility and age show ORs of 6.86 and 3.02, respectively. According to the Phi value (p<0.001), the strength of the association between the presence of a discharge note and the medical department facility is higher than that for age (Ф=0.321).

### Association of patient and transfer characteristics and discharge note

Table 3 shows the results of a multivariate logistic regression model examining factors associated with the presence of a discharge note, including age, gender, medical department facility, and length of hospital stay. All the characteristics included in the model were significantly associated with the presence of a discharge note. Controlled for the other factors in the model, medical department facility had the strongest predictive value for the presence of a discharge note; a discharge note was sent to HC 9.54 times more often following hospitalization in a geriatric unit than following hospitalization in a non-geriatric unit. The next most powerful predictor was age; transfers of patients between the ages of 85–95 were 4.10 times more likely to include nursing discharge notes than those of patients aged 70–84. The third most powerful predictor was length of hospital stay; hospitalizations for 7 days or more included discharge notes 3.02 times more often than hospitalizations that lasted 1–6 days. Finally, gender had a predictive value in our model; transfers of male patients were 2.92 times more likely to include nursing discharge notes than transfers of female patients.

## Discussion

To our knowledge, this is the first published study to evaluate written communication from HC nurses to hospital at admission and the reciprocal written communication from hospital nurses to HC nurses at discharge, during the transfer of older people. Since the entire communication loop was followed for each patient, the study gives us an opportunity to get an overall picture of the information flow.

There was only one instance out of the 102 patients in our sample, in which a nursing transfer document was exchanged both at admission and at discharge. This is in large contrast to the finding of Doare et al. [50], where 37% (n=773) of the patient transfers included a paired GP referral letter and discharge summary. Also when looking only at the communication at admission, our findings differ sharply from previous studies that have addressed the communication between physicians [46, 50]. Transfer documents were identified for almost 69% of the patients at discharge in our study, which, indeed, contrasts highly with the prevalence of documents at admission. On the other hand, we have to face the fact that almost one-third of the patients still went home to HC nurses who did not simultaneously receive nursing documents from the hospital. Our finding is almost in line with previous studies reporting the communication between physicians [47], but is in contrast to some earlier studies of nurses’ information exchange where the prevalence of discharge notes has been reported as low as 5% [45]. The study of Hellesø et al. [44], which is most relevant to this current study because it included only patients discharged to HC, reported a prevalence of 58%.

The finding that assignment to a geriatric unit is a significant predictor for the presence of a nursing discharge note is in agreement with Boockvar and Burack [51], although their findings were based on a survey mailed to administrators and staff at nursing homes. Transfers from geriatric units were more than nine times more likely to include a discharge note compared to the non-geriatric unit in our study. This may be explained by the presence of more skilled and motivated nurses in geriatric in-hospital care [54]. Another explanation could be that transitional care, including communication between providers, is recognized as a core competency in geriatric care [55, 56]. As far as we are aware, there have been no previous studies of relationships between the prevalence of transfer documents and age, gender, and length of hospital stay; thus we are not able to compare our results with previous findings. The fact that transfers of patients in the oldest age group were roughly four times more likely to include nursing discharge notes was a pleasant discovery, as continuity of care is particularly important for the oldest of the old. Transfers with the longest duration of hospital stay were three times more likely to include discharge notes compared to hospitalizations of shorter durations. This may be explained by the nurses’ familiarity with the patients, as providers have reported lack of familiarity with patients to be an important barrier to communication between organizational levels [57]. Finally, there may be many explanations for the increased likelihood of male patient transfers to include nursing discharge notes in our study, for example, that men do not usually talk much about their health condition.

In our study, the hospital nurses seem to be more capable of delivering information about the patients during transfer than the HC nurses. This may appear paradoxical considering the patients short length of stay at the hospital vs. perhaps months or years as an HC recipient. However, prolonged HC does not necessarily imply that the HC nurses are more familiar with their patients and updated on the patients’ current status. The frequencies of assistance provided by HC vary considerably. While some patients get assistance several times a day, other patients may receive, for example, only medical assistance once every other week. In addition, a high number of nursing staff is often encountered in the care of the single patient, which can mean that months pass before a particular nurse sees the same patient. This lack of relational continuity, and lack of knowledge of the patient, could be compensated by available nursing documentation. However, studies have shown that the documentation in HC can be incomplete [58] and even inaccessible [59]. An explanation for this may be a lack of motivation to spend time on information management at the expense of the patients [60]. Nurses have described that prioritizing is unavoidable in HC due to the predefined schedule including lists specifying the given timeframe for their particular tasks [61]. Since New Public Management became the model for organizational structure in Norwegian municipalities, the HC services have become more divided and fragmented due to a change in the relationship between the administrative function and the staff [62]. While the managers, in the role as purchaser, are to be the driving forces for increased productivity and efficiency, the providers have to deliver care within strict standards and limited autonomy. As HC, in contrast with inpatient care, is not limited by bed spaces or contained by walls, it has been likened to “a ward without wall” [60] where the care environment is constantly expanding. Thus, the nurses cannot limit the number of patients, and have to prioritize even though they consider it as a threat to the quality of care, including information management. Although it has been emphasized that nurses in outpatient care need extraordinary communication skills to reach concordance in outpatient care planning [63], the nurses have expressed lack of support from their leaders to facilitate enough time for documentation and planning [59].

The fact that patients are likely to be transferred urgently from HC to hospital suggests an obvious explanation for the low prevalence of transfer documents at admission: It goes without saying that patients admitted to a hospital are supposed to be discharged sooner or later, thus, the hospital nurses expect a transfer, and they may plan for discharge from the very day of admission. For the HC nurses, on the other hand, an urgent hospitalization is, in its nature, unexpected and hard to plan. Lack of documentation has been reported as a major issue in acute situations in HC [59]. In addition, lack of time [64], a large physical distance between patients and their records [59], and nurses’ problems with reaching one another on the phone [63], complicate the gathering and transfer of information even more. Sending the transfer document at a later time could solve the problems with transferring information at admission. However, this request that the particular nurse is on duty or that the information about the admission is handed over. Standardized procedures and checklists may help as a cognitive aid or organizational tool to improve the transfer of information [28, 47].

Lack of transfer documents from HC may also be explained by the fact that providers in primary care in some cases are unaware that their patient is hospitalized [49]. His/her GP or perhaps an on-call physician who may not even know that the patient is an HC recipient may admit the patient. If, in the latter case, the patient lives alone and accompanied by any relatives, the issue of notifying the HC largely depends on the patient’s condition and ability to tell. A better collaboration between GP and HC has been emphasized to ensure safe patient transitions between primary and secondary care [2].

The low prevalence of nursing transfer documents, and particularly admission notes, raises a concern about the continuity of care to older people transferred between HC and hospital. Based on the framework of Haggerty et al. [31], one can assume that the comprehensiveness and accuracy of the transfer documents have an impact on the informational continuity, and that the extent of planned interventions and described responsibilities for follow-up care in the documents influence the management continuity. However, to improve continuity to older patients in transfer, there is a need not only to document adequate information, but also to transfer this information at admission and at discharge. Both nurses and nursing managers have important roles in this process. While nurses must strive to transfer accurate patient information at the right place and at the right time, the managers must facilitate this by providing appropriate guidelines and standards, and adequate personnel and resources.

Finally, our data cannot tell us whether other methods of information dissemination have been used at admission. Several studies have shown that information transfer between hospital and primary care largely occurs through informal means of communication [43, 59, 60], thus, the continuity may have been maintained by verbal communication (e.g., telephone) in our sample. Further research should take into account both the written and verbal communication during transfer, for example, by using observational methods.

### Study limitations

A potential limitation of the present study is the fact that the sets of transfer documents were retrieved from the patient records at the hospital. We only had the opportunity to identify transfer documents that were received by and sent from the hospital, thus, we had no way to verify whether HC had generated and sent documents that might have been lost on the way. The fact that participant recruitment was done at the hospital may have biased the sample. If the recruitment had been done by the HC, the sample may have included more patients with admission notes. The use of consecutive convenience sampling and the fact that not all patients in the actual hospital ward could be included in the study during the data collection period can also be regarded as limitations to the validity and generalizability of the results. Due to the requirements of informed consent and voluntary participation, patients who had cognitive impairments (e.g., dementia) were excluded. Thus, the results of our study must be viewed in light of the study group, i.e., older patients who are consent competent. The low prevalence of patients with congestive heart failure (CHF) (2.9%) in our sample may appear surprising considering that this is a common disease in elderly patients. An explanation could be that the current hospital has a special unit for patients with heart diseases, and this unit is not included in our study. Another reason may be the fact that we only addressed the main admission diagnosis, and CHF is often present as a secondary diagnosis. As it was not permissible to collect data from those patients who did not volunteer for the study (cf. The Committee for Medical and Health Research Ethics), we had no opportunity to perform drop-out analyses. However, only 9 patients who fulfilled the inclusion criteria declined to participate in the study. The small sample size limits the generalization of our findings. However, 70 cases satisfied the recommended sample size (10 cases per independent variable) for logistic regression [65]. The fact that our model explained 30.7% of the variation in the presence of discharge notes suggests that other patient and transfer characteristics were likely to be of importance. However, data relating to other characteristics (e.g., function status, off-hours transfers, and frequency of assistance provided by HC) were unavailable to us; thus, we could not further evaluate the possible impact of these factors.

## Conclusion

The low prevalence of nursing transfer documents constitutes a challenge to the continuity of care for hospitalized HC patients. Patient and transfer characteristics may impact the nurses’ propensity to exchange patient information. These findings emphasize the need for nurses and managers to improve the exchange of written information. While nurses must strive to transfer accurate patient information at the right place and at the right time, the managers must facilitate this by providing appropriate guidelines and standards, as well as adequate personnel and resources.

## Reviewers

**Isabelle Brault,** inf., PhD, Professeure adjointe, Faculté des sciences infirmières, Pavillon Marguerite-d’Youville, 2375, chemin Côte-Ste-Catherine, bureau 5092, Montréal (Québec), H3T 1A8 Canada

**Abraham Aizer Brody,** RN, PhD, GNP-BC, Assistant Professor, NYU College of Nursing, USA

**Ragnhild Hellesø**, RN, PhD, Associate Professor, Faculty of Medicine, Institute of Health and Society, Department of Nursing and Health Sciences, University of Oslo, P.O. Box 1153 Blindern, No-0318 Oslo, Norway

**Paul Stolee**, PhD, School of Public Health and Health Systems, University of Waterloo, Waterloo, Ontario, Canada

## Figures and Tables

**Table 1.**
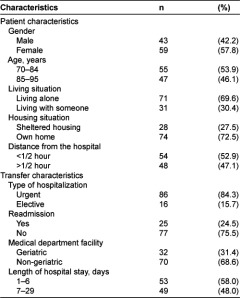
Characteristics of the sample (n=102).

**Table 2:**
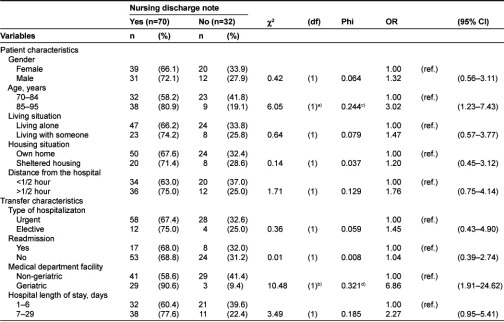
Relation between presence of nursing discharge note and background variables (n=102).

^a)^ Pearson χ^2^ <0.01. ^b)^Pearson χ^2^<0.001. ^c)^ Phi<0.01. ^d)^ Phi<0.001.

**Table 3:**
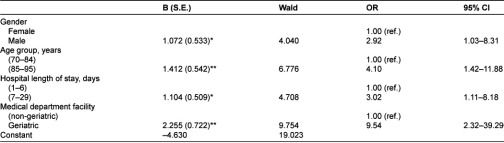
Logistic regression model.

R^2^=(Nagelkerke).307.

Hosmer and Lemeshow model goodness of fit p=0.751.

Model χ^2^=25.147 (p<0.001),*p<0.05, **p<0.01.
